# Abnormal gut microbiota composition contributes to cognitive dysfunction in streptozotocin-induced diabetic mice

**DOI:** 10.18632/aging.101978

**Published:** 2019-05-23

**Authors:** Fan Yu, Wei Han, Gaofeng Zhan, Shan Li, Shoukui Xiang, Bin Zhu, Xiaohong Jiang, Ling Yang, Ailin Luo, Fei Hua, Chun Yang

**Affiliations:** 1 Department of Endocrinology, The Third Affiliated Hospital of Soochow University, Changzhou 213003, China; 2 Department of Neurosurgery, The Third Affiliated Hospital of Soochow University, Changzhou 213003, China; 3 Department of Anesthesiology, Tongji Hospital, Tongji Medical College, Huazhong University of Science and Technology, Wuhan 430030, China; 4 Department of Critical Care Medicine, The Third Affiliated Hospital of Soochow University, Changzhou 213003, China; 5 Department of Cardiology, The Third Affiliated Hospital of Soochow University, Changzhou 213003, China

**Keywords:** diabetes, cognitive dysfunction, gut microbiota, streptozotocin, hierarchical cluster analysis

## Abstract

Both diabetes and Alzheimer’s disease are age-related disorders, and numerous studies have demonstrated that patients with diabetes are at an increased risk of cognitive dysfunction (CD) and Alzheimer’s disease, suggesting shared or interacting pathomechanisms. The present study investigated the role of abnormal gut microbiota in diabetes-induced CD and the potential underlying mechanisms. An intraperitoneal injection of streptozotocin administered for 5 consecutive days was used for establishing a diabetic animal model. Hierarchical cluster analysis of Morris water maze (MWM) performance indices (escape latency and target quadrant crossing) was adopted to classify the diabetic model mice into CD and Non-CD phenotypes. Both β-diversity and relative abundance of several gut bacteria significantly differed between the CD and Non-CD groups. Further, fecal bacteria transplantation from Non-CD mice, but not from CD mice, into the gut of pseudo-germ-free mice significantly improved host MWM performance, an effect associated with alterations in β-diversity and relative abundance of host gut bacteria. Collectively, these findings suggest that abnormal gut microbiota composition contributes to the onset of diabetes-induced CD and that improving gut microbiota composition is a potential therapeutic strategy for diabetes and related comorbidities.

## INTRODUCTION

The incidences of both diabetes and Alzheimer’s disease increase with age [1, 2]. Although the complete pathogenic mechanisms underlying diabetes and Alzheimer’s disease remain unclear, there is emerging evidence that certain neuropathological processes are common to both disorders; indeed, both are associated with cognitive dysfunction (CD) [3, 4]. However, currently available treatments for diabetes and Alzheimer’s disease are mainly symptomatic and do not target the underlying pathogenesis [5–7].

Further corroborating a link between diabetes and Alzheimer’s disease, it has been reported that patients with diabetes are at a higher risk of neurological disorders [8], with mild CD being the most common afflicting up to 19% of all such patients [9]. Therefore, exploring the pathogenesis of CD caused by diabetes and developing effective treatment strategies are critical for improving the quality of life and functional independence of patients with diabetes. Moreover, these patients are at a higher risk of progressing from CD to Alzheimer’s disease; therefore, identifying the pathogenic interactions between these disorders may yield novel therapeutic strategies for both disorders [10, 11]. Accumulating evidence suggests that the pathogenesis of Alzheimer’s disease involves abnormal brain glucose metabolism and insulin signaling as well as the deposition of cytotoxic amyloid β (Aβ) [10, 12, 13]. Furthermore, type 3 diabetes is currently considered a form of Alzheimer’s disease resulting from brain insulin resistance [14].

Gut microbiota strongly influences brain function and energy metabolism [15]. Our previous study has demonstrated a strong association between CD in SAMP8 mice and abnormal gut microbiota composition [16]. In addition, CD in aged mice following surgery and anesthesia may be associated with altered gut microbiota composition [17]. Furthermore, gut microbiota dysbiosis and increased intestinal permeability have been observed in patients with diabetes [18, 19]. Based on these findings, we speculated that CD caused by diabetes may be related to abnormal gut microbiota composition.

Considering the potential role of gut microbiota in diabetes-induced CD, we used 16S rRNA gene sequencing to compare gut bacterial composition between CD and Non-CD phenotypes of diabetes. Furthermore, we examined the effects of fecal bacteria transplantation from diabetes-induced CD and Non-CD phenotypes on spatial memory and gut microbiota composition of host pseudo-germ-free mice.

## RESULTS

### Differences in spatial learning and memory among control, diabetic CD, and diabetic Non-CD mice

**Figure 1:**
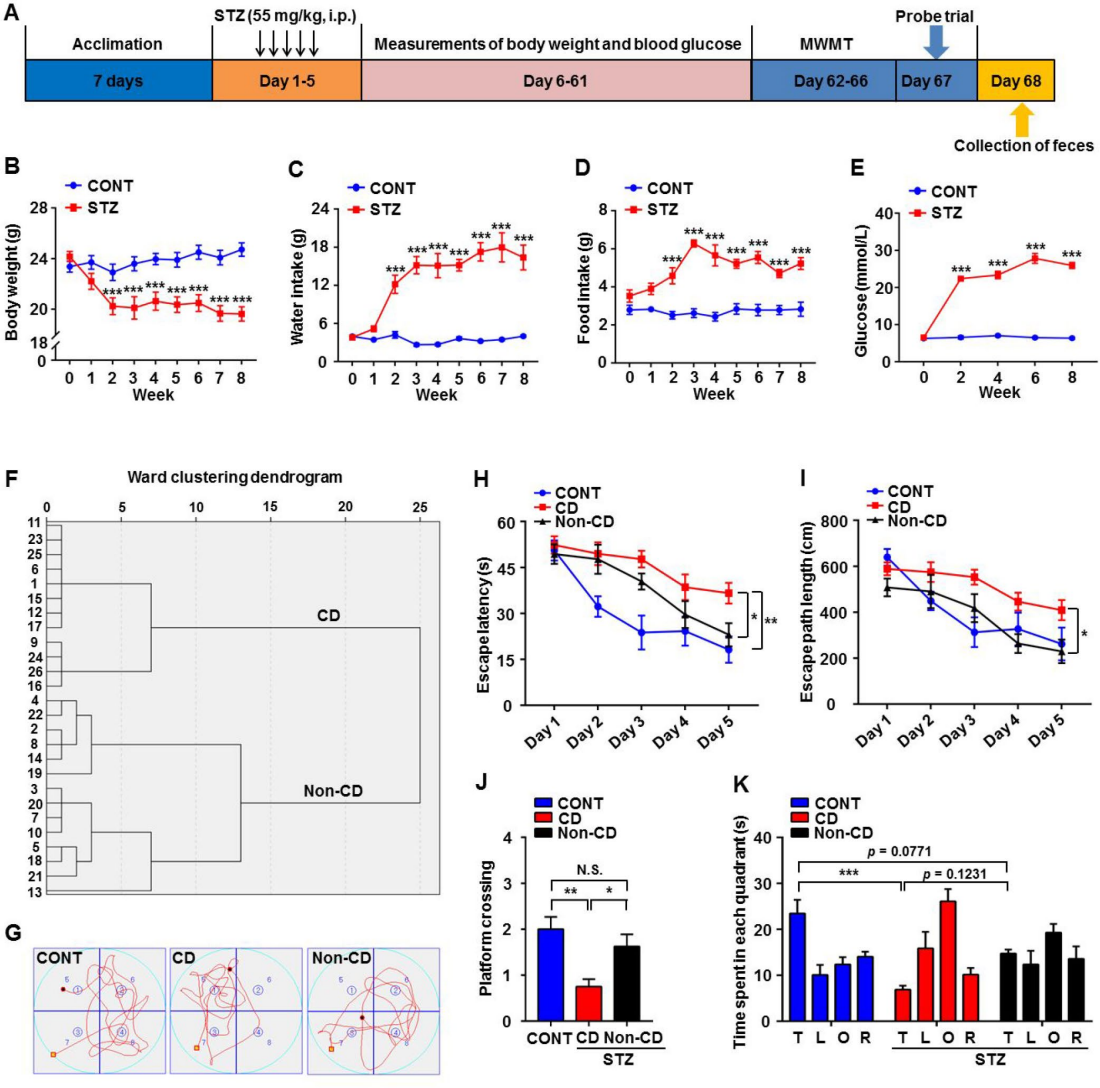
**Comparisons of Morris water maze performance among control (CONT), diabetic cognitive dysfunction (CD), and diabetic Non-CD mouse groups.** (**A**) The schedule of the present study. At 7 days after acclimation, mice were intraperitoneally injected with STZ (55 mg/kg) for 5 consecutive days to induce diabetes or with vehicle as a control. Body weight, water and food intake, and blood glucose levels were measured from day 6 to 61. Mice were scheduled for MWM training (4 trials per day) from day 62 to 66 post-STZ, and the probe trial was performed on day 67. On day 68, fecal samples were collected for 16S rRNA gene sequencing. (**B**) Body weight (two-way ANOVA; Time: F_8,56_ = 8.446, *p* < 0.001; Group: F_1,7_ = 20.46, *p* < 0.01; Time × Group Interaction: F_8,56_ = 12.34, *p* < 0.001). (**C**) Water intake (two-way ANOVA; Time: F_8,56_ = 17.48, *p* < 0.001; Group: F_1,7_ = 105.1, *p* < 0.001; Interaction: F_8,56_ = 19.67, *p* < 0.001). (**D**) Food intake (two-way ANOVA; Time: F_8,56_ = 5.254, *p* < 0.001; Group: F_1,7_ = 108.4, *p* < 0.001; Interaction: F_8,56_ = 5.755, *p* < 0.001). (**E**) Blood glucose levels (two-way ANOVA; Time: F_4,28_ = 66.98, *p* < 0.001; Group: F_1,7_ = 2376, *p* < 0.001; Interaction: F_4,28_ = 79.15, *p* < 0.001). (**F**) Dendrogram of hierarchical clustering analysis. A total of 26 mice confirmed as diabetic following STZ injection were divided into CD and Non-CD groups according to MWM performance indices using hierarchical clustering analysis. (**G**) Representative trace graphs of CONT, CD, and Non-CD group swim paths in the MWM. (**H**) Escape latency (two-way ANOVA; Time: F_4,28_ = 23.09, *p* < 0.001; Group: F_2,14_ = 14.84, *p* < 0.001; Interaction: F_8,56_ = 1.57, *p* > 0.05). (**I**) Escape path length (two-way ANOVA; Time: F_4,28_ = 14.36, *p* < 0.001; Group: F_2,14_ = 15.74, *p* < 0.001; Interaction: F_8,56_ = 1.292, *p* > 0.05). (**J**) Platform crossings (one-way ANOVA; F_2,21_ = 7.373, *p* < 0.01). (**K**) Time spent in each quadrant (two-way ANOVA; Time: F_3,21_ = 5.917, *p* < 0.01; Group: F_2,14_ = 0.9345, *p* > 0.05; Interaction: F_6,42_ = 5.618, *p* < 0.001). Data are shown as mean ± SEM (n = 8−10 mice/group). *P < 0.05, **P < 0.01 or ***P < 0.001. ANOVA: analysis of variance; CD: cognitive dysfunction; CONT: control; MWM: Morris water maze; N.S.: not significant; SEM: standard error of the mean; STZ: streptozotocin.

An intraperitoneal injection of 55 mg/kg strepozotocin (STZ) for 5 consecutive days was administered to establish a type 1 diabetes model (Figure 1A). Successful induction of diabetes was confirmed following the final STZ dose (Figure 1B–1E) by comparing body weight, water and food intake, and blood glucose levels between the diabetes model and age-matched control mice. Body weight was significantly lower in STZ-treated mice (Figure 1B). Moreover, at 2 weeks after final STZ exposure, model mice exhibited significantly higher water and food intake as well as higher blood glucose levels (Figure 1C–1E). After 2 months, a total of 26 STZ-treated mice confirmed as diabetic were divided into Non-CD and CD groups according to the hierarchical clustering analysis of the Morris water maze (MWM) performance (Figure 1F). A notable difference of swimming traces in MWMT was represented among CONT, CD, and Non-CD groups (Figure 1G). Both hidden platform escape latency and path length in the MWM training phase were significantly higher in the diabetes model group compared to the control group, indicating deficient spatial learning. However, in the diabetes model group, performance was stratified into a CD group demonstrating significantly higher escape latencies and path lengths and a Non-CD group with relatively normal performance indices (Figure 1H and 1I). Furthermore, in the probe trial, the number of platform crossings was significantly lower in CD mice than control or Non-CD mice (Figure 1J). Moreover, in the target quadrant, CD mice spent significantly lesser time than control mice as well as numerically lesser time than Non-CD mice, although this difference was not significant (Figure 1K). Therefore, a subpopulation of the diabetic mice (CD group) demonstrated deficient spatial memory.

### Differences in gut microbiota profile among control, CD, and Non-CD mice

**Figure 2:**
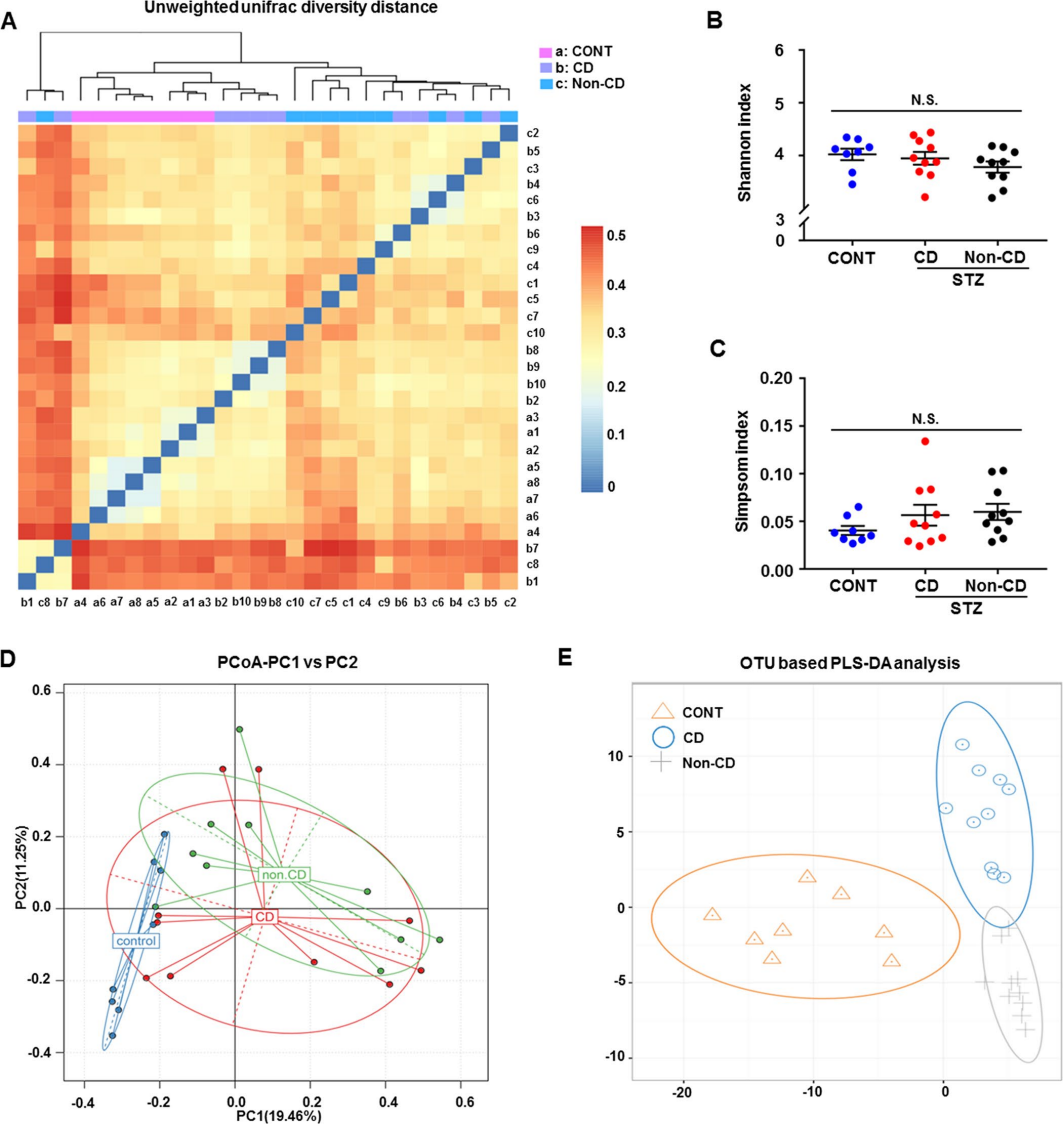
**Differences in gut microbiota profiles among CONT, CD, and Non-CD mice.** (**A**) Unweighted unifrac diversity distance. (**B**) Shannon index (one-way ANOVA; F_2,25_ = 1.17, *p* > 0.05). (**C**) Simpson index (one-way ANOVA; F_2,25_ = 1.272, *p* > 0.05). (**D**) PCoA analysis of gut bacteria (PC1 versus PC2). (**E**) PLS-DA analysis of gut bacteria. The α-diversity is shown as mean ± SEM (n = 8−10 individual fecal samples/group). ANOVA: analysis of variance; CD: cognitive dysfunction; CONT: control; N.S.: not significant; PCoA: principal coordinate analysis; PLS-DA: partial least squares discrimination analysis; SEM: standard error of the mean.

A plot of unweighted unifrac diversity distance suggested marked differences in gut microbiota composition among groups (Figure 2A). Although Shannon and Simpson indices failed to show such a difference (Figure 2B and 2C), a partial least squares discrimination analysis (PLS-DA) and principal coordinate analysis (PCoA) both yielded well separated positions among groups (Figure 2D and 2E). Therefore, it is likely that gut microbiota composition is distinct among groups.

### Gut microbiota composition at phylum, class, order, family, genus, and species levels among control, CD, and Non-CD mice

**Figure 3:**
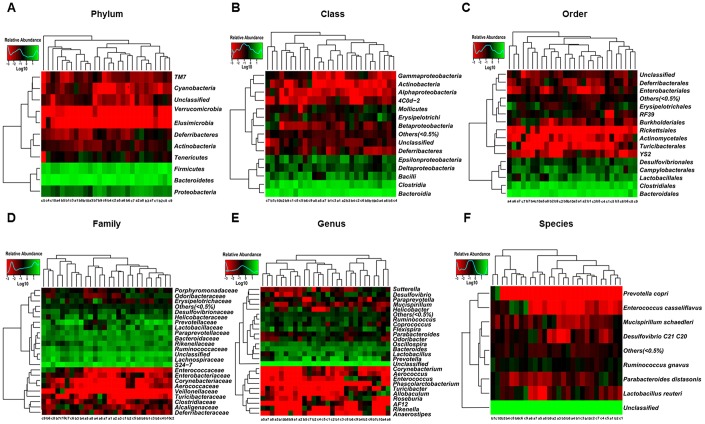
**Heatmaps of gut microbiota composition at phylum, class, order, family, genus, and species levels for CONT, CD, and Non-CD mice.** (**A**) Heatmap (phylum level). (**B**) Heatmap (class level). (**C**) Heatmap (order level). (**D**) Heatmap (family level). (**E**) Heatmap (genus level). (**F**) Heatmap (species level).

**Figure 4:**
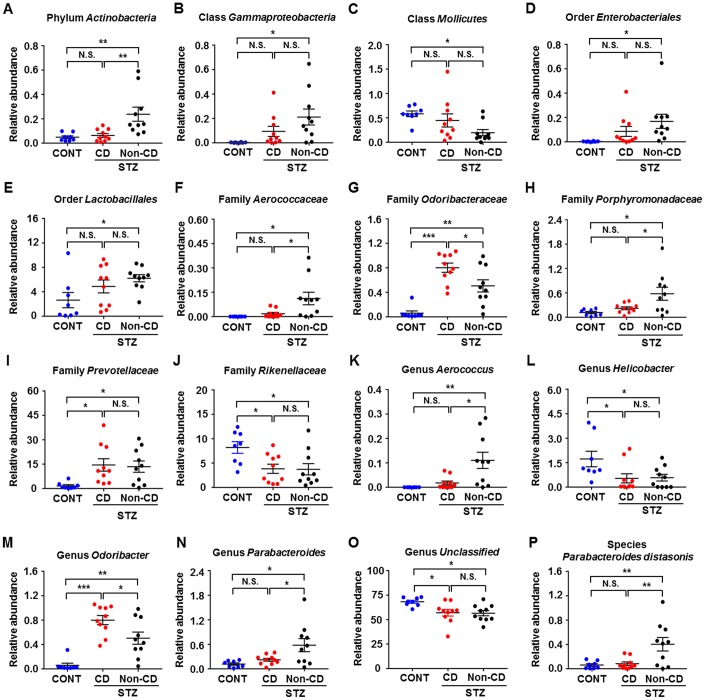
**Differences in the relative abundance of various gut microbes among CONT, CD, and Non-CD mice.** (**A**–**P**) Relative abundances of (**A**) phylum *Actinobacteria* (one-way ANOVA; F_2,25_ = 7.958, *p* < 0.01), (**B**) class *Gammaproteobacteria* (one-way ANOVA; F_2,25_ = 4.597, *p* < 0.05), (**C**) class *Mollicutes* (one-way ANOVA; F_2,25_ = 4.035, *p* < 0.05), (**D**) order *Enterobacteriales* (one-way ANOVA; F_2,25_ = 3.385, *p* = 0.05), (**E**) order *Lactobacillales* (one-way ANOVA; F_2,25_ = 3.277, *p* > 0.05), (**F**) family *Aerococcaceae* (one-way ANOVA; F_2,25_ = 6.019, *p* < 0.01), (**G**) family *Odoribacteraceae* (one-way ANOVA; F_2,25_ = 20.67, *p* < 0.001), (**H**) family *Porphyromonadaceae* (one-way ANOVA; F_2,25_ = 5.597, *p* < 0.01), (**I**) family *Prevotellaceae* (one-way ANOVA; F_2,25_ = 4.528, *p* < 0.05), (**J**) family *Rikenellaceae* (one-way ANOVA; F_2,25_ = 4.938, *p* < 0.05), (**K**) genus *Aerococcus* (one-way ANOVA; F_2,25_ = 7.863, *p* < 0.01), (**L**) genus *Helicobacter* (one-way ANOVA; F_2,25_ = 4.135, *p* < 0.05), (**M**) genus *Odoribacter* (one-way ANOVA; F_2,25_ = 20.78, *p* < 0.001), (**N**) genus *Parabacteroides* (one-way ANOVA; F_2,25_ = 5.597, *p* < 0.01), (**O**) genus *Unclassified* (one-way ANOVA; F_2,25_ = 5.114, *p* < 0.05), and (**P**) species *Parabacteroides distasonis* (one-way ANOVA; F_2,25_ = 7.235, *p* < 0.01). Data are shown as mean ± SEM (n = 8−10 individual fecal samples/group). *P < 0.05, **P < 0.01 or ***P < 0.001. ANOVA: analysis of variance; CD: cognitive dysfunction; CONT: control; N.S.: not significant; SEM: standard error of the mean.

The heat maps of the gut microbiota composition at the phylum, class, order, family, genus, and species levels show specific differences among control, CD, and Non-CD groups (Figure 3A–3F). 16S rRNA gene sequencing revealed that a total of 16 gut bacteria at 6 phylogenetic levels (phylum, class, order, family, genus, and species) significantly differed among fecal samples from the three groups (Figure 4A–4P). The relative abundances of family *Odoribacteraceae*, family *Prevotellaceae*, and genus *Odoribacter* were significantly higher in the CD group than control group (Figure 4G, 4I, and 4M), whereas the relative abundances of family *Rikenellaceae*, genus *Helicobacter*, and genus *Unclassified* were significant lower in the CD group than the control group (Figure 4J, 4L, and 4O). The relative abundances of six bacterial species were significantly lower in the CD group than the Non-CD group (Figure 4A, 4F, 4H, 4K, 4N, and 4P), whereas the relative abundances of two species were significantly higher in the CD group than the Non-CD group (Figure 4G and 4M). There were no significant differences in eight gut bacteria between CD and Non-CD groups (Figure 4B–4E, 4I, 4J, 4L, and 4O).

### Correlations between spatial memory and specific gut bacteria

**Figure 5:**
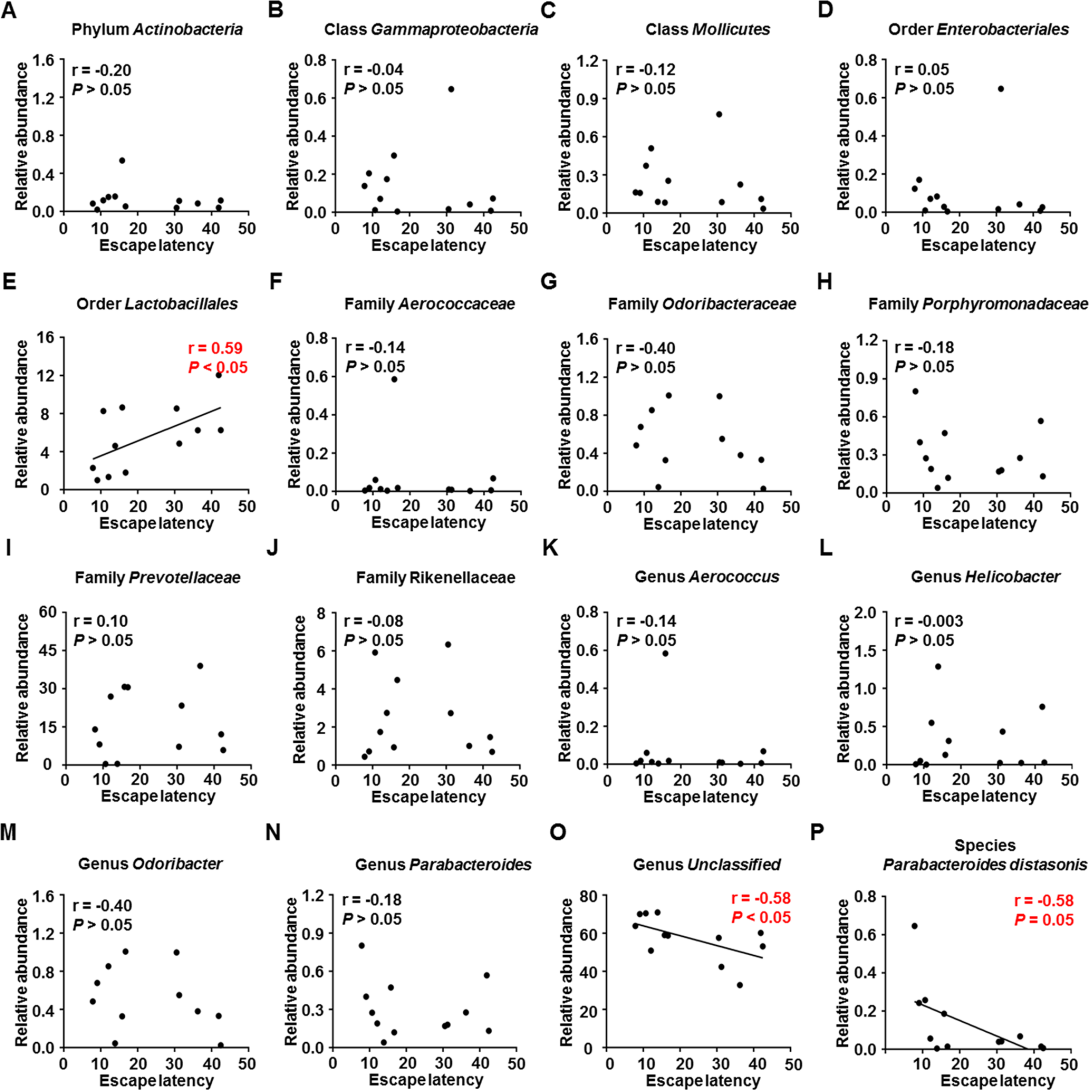
**Correlations between MWM escape latency and relative abundance of various gut microbes (N = 12).** (**A**) Phylum *Actinobacteria* (r = −0.20, *P* > 0.05). (**B**) Class *Gammaproteobacteria* (r = −0.04, *P* > 0.05). (**C**) Class *Mollicutes* (r = −0.12, *P* > 0.05). (**D**) Order *Enterobacteriales* (r = 0.05, *P* > 0.05). (**E**) Order *Lactobacillales* (r = 0.59, *P* < 0.05). (**F**) Family *Aerococcaceae* (r = −0.14, *P* > 0.05). (**G**) Family *Odoribacteraceae* (r = −0.40, *P* > 0.05). (**H**) Family *Porphyromonadaceae* (r = −0.18, *P* > 0.05). (**I**) Family *Prevotellaceae* (r = 0.10, *P* > 0.05). (**J**) Family *Rikenellaceae* (r = −0.08, *P* > 0.05). (**K**) Genus *Aerococcus* (r = −0.14, *P* > 0.05). (**L**) Genus *Helicobacter* (r = −0.003, *P* > 0.05). (**M**) Genus *Odoribacter* (r = −0.40, *P* > 0.05). (**N**) Genus *Parabacteroides* (r = −0.18, *P* > 0.05). (**O**) Genus *Unclassified* (r = −0.58, *P* < 0.05). (**P**) Species *Parabacteroides distasonis* (r = −0.58, *P* = 0.05). MWM: Morris water maze.

Escape latency in the MWM training trials was adopted as a measure of spatial learning to evaluate the effects of gut microbiota composition on cognitive function. Correlation analysis (Figure 5A–5P) revealed a positive association between escape latency and the relative abundance of order *Lactobacillales* (Figure 5E) and negative associations between escape latency and the relative abundances of genus *Unclassified* and species *Parabacteroides distasonis* (Figure 5O and 5P). Therefore, the relative abundances of specific gut bacteria can either positively or negatively influence spatial learning ability.

### Evaluation of gut bacteria for the diagnosis of diabetes-induced CD using receiver operating characteristic curve analysis

**Figure 6:**
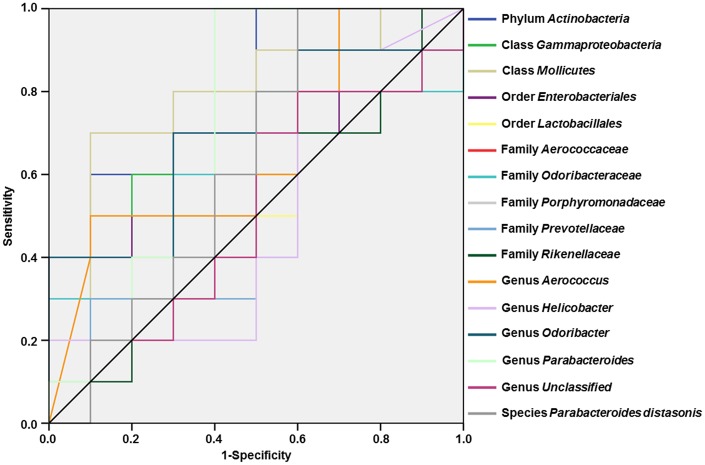
**ROC curves of various gut microbes for the diagnosis of diabetes-induced cognitive dysfunction.** (**A**) Phylum *Actinobacteria* (AUC = 0.800). (**B**) Class *Gammaproteobacteria* (AUC = 0.680). (**C**) Class *Mollicutes* (AUC = 0.800). (**D**) Order *Enterobacteriales* (AUC = 0.640). (**E**) Order *Lactobacillales* (AUC = 0.600). (**F**) Family *Aerococcaceae* (AUC = 0.660). (**G**) Family *Odoribacteraceae* (AUC = 0.600). (**H**) Family *Porphyromonadaceae* (AUC = 0.710). (**I**) Family *Prevotellaceae* (AUC = 0.520). (**J**) Family *Rikenellaceae* (AUC = 0.520). (**K**) Genus *Aerococcus* (AUC = 0.660). (**L**) Genus *Helicobacter* (AUC = 0.510). (**M**) Genus *Odoribacter* (AUC = 0.700). (**N**) Genus *Parabacteroides* (AUC = 0.710). (**O**) Genus *Unclassified* (AUC = 0.510). (**P**) Species *Parabacteroides distasonis* (AUC = 0.630). AUC: area under the curve; ROC: receiver operating characteristic.

Receiver operating characteristic (ROC) curves were constructed to assess the ability of specific gut bacteria to identify diabetes-induced CD (Figure 6). The best cutoff values, sensitivity, specificity, and accuracy as well as the positive and negative predictive values of gut bacteria for the diagnosis of diabetes-induced CD are summarized in Table 1.

**Table 1:** Evaluation of various gut microbes for diagnosis of diabetes-induced cognitive dysfunction.

**Evaluation index**	**Cut-off value**	**Sensitivity**	**Specificity**	**Positive predictive value**	**Negative predictive value**	**Accuracy**
Phylum *Actinobacteria*, (n)	0.0528	60% (6/10)	90% (9/10)	85.7% (6/7)	69.2% (9/13)	75% (15/20)
Class *Gammaproteobacteria*, (n)	0.0720	70% (7/10)	70% (7/10)	70% (7/10)	70% (7/10)	70% (14/20)
Class *Mollicutes*, (n)	0.1753	70% (7/10)	80% (8/10)	77.8% (7/9)	72.7% (8/11)	75% (15/20)
Order *Enterobacteriales*, (n)	0.0408	70% (7/10)	70% (7/10)	70% (7/10)	70% (7/10)	70% (14/20)
Order *Lactobacillales*, (n)	8.5336	90% (9/10)	40% (4/10)	60% (9/15)	80% (4/5)	65% (13/20)
Family *Aerococcaceae*, (n)	0.0020	50% (5/10)	90% (9/10)	83.3% (5/6)	64.3% (9/14)	70% (14/20)
Family *Odoribacteraceae*, (n)	0.9890	40% (4/10)	100% (10/10)	100% (4/4)	62.5% (10/16)	70% (14/20)
Family *Porphyromonadaceae*, (n)	0.4001	100% (10/10)	60% (6/10)	71.4% (10/14)	100% (6/6)	80% (16/20)
Family *Prevotellaceae*, (n)	5.5134	80% (8/10)	40% (4/10)	57.1% (8/14)	66.7% (4/6)	60% (12/20)
Family *Rikenellaceae*, (n)	2.7392	50% (5/10)	70% (7/10)	62.5% (5/8)	58.3% (7/12)	60% (12/20)
Genus *Aerococcus*, (n)	0.0020	50% (5/10)	90% (9/10)	83.3% (5/6)	64.3% (9/14)	70% (14/20)
Genus *Helicobacter*, (n)	0.0082	90% (9/10)	40% (4/10)	60% (9/15)	80% (4/5)	65% (13/20)
Genus *Odoribacter*, (n)	0.9890	40% (4/10)	100% (10/10)	100% (4/4)	62.5% (10/16)	70% (14/20)
Genus *Parabacteroides*, (n)	0.4001	100% (10/10)	60% (6/10)	71.4% (10/14)	100% (6/6)	80% (16/20)
Genus *Unclassified*, (n)	50.9319	80% (8/10)	40% (4/10)	57.14% (8/14)	66.7% (4/6)	60% (12/20)
Species *Parabacteroides distasonis*, (n)	0.2573	100% (10/10)	40% (4/10)	62.5% (10/16)	100% (4/4)	70% (14/20)

### Effects of CD and Non-CD gut microbiota transplantation on spatial learning and memory in pseudo-germ-free mice

**Figure 7:**
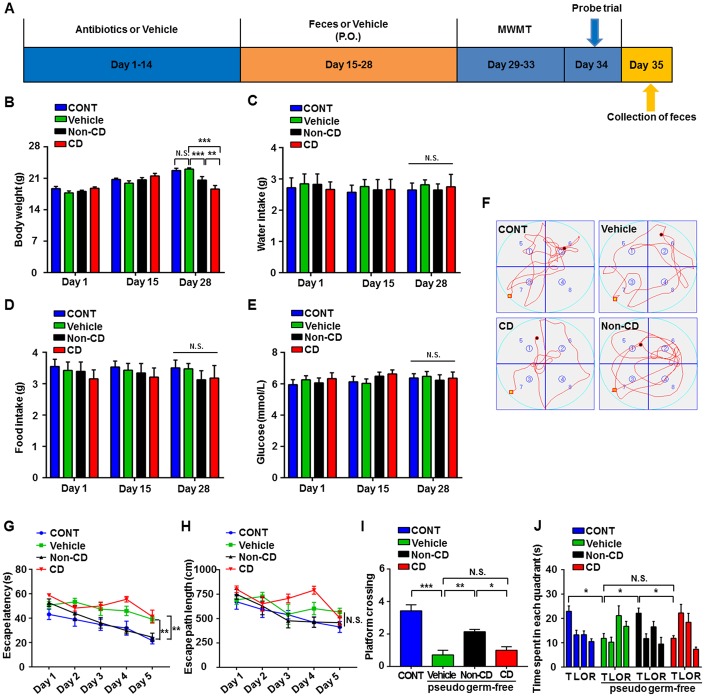
**Effects of fecal microbiota transplantation from CD and Non-CD mice on MWM performance by pseudo-germ-free mice.** (**A**) Schedule for evaluation of MWM performance by pseudo-germ-free (host) mice transplanted with gut bacteria from diabetic mice. Host mice were treated with large doses of antibiotic solution for 14 consecutive days, and then orally treated with fecal microbiota from CD or Non-CD mice. MWM training trials were conducted from day 29 to 33, and the probe trial was performed on day 34. On day 35, fecal samples were collected for 16S rRNA gene sequencing. (**B**) Body weight (two-way ANOVA; Time: F_2,12_ = 76.89, *p* < 0.001; Group: F_3,18_ = 1.455, *p* > 0.05; Interaction: F_6,36_ = 13.85, *p* < 0.001). (**C**) Water intake (two-way ANOVA; Time: F_2,10_ = 1.016, *p* > 0.05; Group: F_3,15_ = 0.074, *p* > 0.05; Interaction: F_6,30_ = 0.133, *p* > 0.05). (**D**) Food intake (two-way ANOVA; Time: F_2,10_ = 0.319, *p* > 0.05; Group: F_3,15_ = 0.367, *p* > 0.05; Interaction: F_6,30_ = 0.445, *p* > 0.05). (**E**) Blood glucose levels (two-way ANOVA; Time: F_2,12_ = 0.433, *p* > 0.05; Group: F_3,18_ = 0.582, *p* > 0.05; Interaction: F_6,36_ = 0.357, *p* > 0.05). (**F**) Representative trace graphs of mouse swim paths in the MWM. (**G**) Escape latency (two-way ANOVA; Time: F_4,24_ = 16.13, *p* < 0.001; Group: F_3,18_ = 16.9, *p* < 0.001; Interaction: F_12,72_ = 1.462, *p* > 0.05). (**H**) Escape path length (two-way ANOVA; Time: F_4,24_ = 10.09, *p* < 0.001; Group: F_3,18_ = 4.763, *p* < 0.05; Interaction: F_12,72_ = 1.679, *p* > 0.05). (**I**) Platform crossings (one-way ANOVA; F_3,24_ = 21.4, *p* < 0.001). (**J**) Time spent in each quadrant (two-way ANOVA; Time: F_3,18_ = 4.359, *p* < 0.05; Group: F_3,18_ = 6.379, *p* < 0.01; Interaction: F_9,54_ = 4.466, *p* < 0.001). Data are shown as mean ± SEM (n = 8 individual samples/group). *P < 0.05, **P < 0.01 or ***P < 0.001. ANOVA: analysis of variance; CD: cognitive dysfunction; CONT: control; MWM: Morris water maze; N.S.: not significant; SEM: standard error of the mean.

A pseudo-germ-free mouse model was established by administering antibiotics at large doses for 14 consecutive days. Gut microbiota from CD and Non-CD mice were transplanted into the gastrointestinal tract of pseudo-germ-free mice through feces for another 14 consecutive days (Figure 7A). There were no significant differences in body weight among pseudo-germ-free control mice, mice receiving vehicle, and mice receiving fecal bacteria from either CD or Non-CD mice on days 1 and 15 post-treatment (Figure 7B). However, by day 28, body weight was significantly lower in mice receiving CD group fecal bacteria compared with group receiving Non-CD mouse fecal bacteria, whereas body weight was significantly higher in the vehicle control group than the group receiving Non-CD mouse fecal bacteria. Therefore, microbiota from diabetic mice appeared to influence host mouse metabolism. Conversely, there were no significant differences in water and food intake as well as blood glucose levels among the four groups on days 1, 15, and 28 (Figure 7C–7E). Representative swimming traces of MWMT were showed in Figure 7F.

Escape latency on training day 5 was significantly longer in pseudo-germ-free mice receiving CD mouse fecal bacteria than those receiving Non-CD mouse fecal bacteria and significantly shorter in the group receiving Non-CD mouse fecal bacteria than the vehicle group (Figure 7G). There was no significant difference in escape path length on day 5 among the four groups (Figure 7H). Therefore, gut microbiota transplanted from Non-CD mice, but not from CD mice, effectively improved the spatial learning performance of host mice. Moreover, in the probe trial, gut microbiota transplantation from Non-CD mice, but not from CD mice, improved the spatial memory performance of host mice as measured by the number of platform crossings and time spent in the target quadrant (Figure 7I and 7J).

### Differences in gut microbiota composition between pseudo-germ-free mice receiving CD or Non-CD mouse fecal bacteria

**Figure 8:**
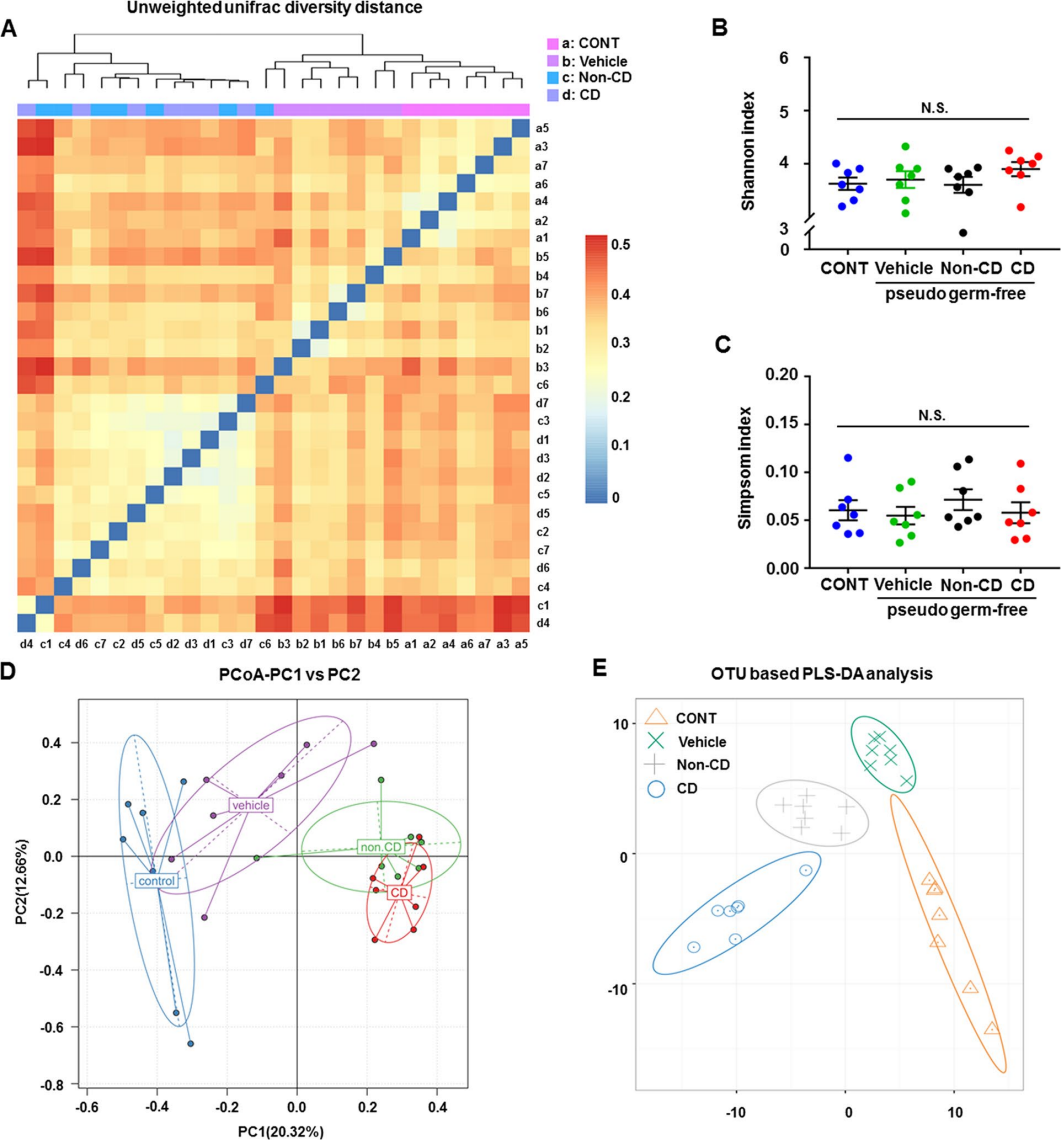
**Changes in the gut microbiota of pseudo-germ-free mice following transplantation from CD and Non-CD diabetic mice.** (**A**) Unweighted unifrac diversity distance. (**B**) Shannon index (one-way ANOVA; F_3,24_ = 0.928, *p* > 0.05). (**C**) Simpson index (one-way ANOVA; F_3,24_ = 0.486, *p* > 0.05). (**D**) PCoA analysis of gut bacteria data (PC1 versus PC2). (**E**) PLS-DA analysis of gut bacteria data. The α-diversity is shown as mean ± SEM (n = 7 individual samples/group). ANOVA: analysis of variance; CD: cognitive dysfunction; CONT: control; N.S.: not significant; PCoA: principal coordinate analysis; PLS-DA: partial least squares discrimination analysis; SEM: standard error of the mean.

A plot of unweighted unifrac diversity distance demonstrated possible differences in gut microbiota composition among control pseudo-germ-free mice and those receiving vehicle, CD mouse fecal bacteria, or Non-CD mouse fecal bacteria (Figure 8A). Although both Shannon and Simpson indices failed to show significant differences among the groups (Figure 8B and 8C), PCoA and PLS-DA plots showed that host mice receiving CD mouse fecal bacteria were well separated from control mice, whereas those receiving Non-CD mouse fecal bacteria were separated from those receiving CD mouse fecal bacteria but not from those receiving vehicle (Figure 8D and 8E). Therefore, it is likely that fecal microbiota transplanted from CD and Non-CD mice induced distinct changes in host gut microbiota.

### Fecal microbiota composition in pseudo-germ-free mice at phylum, class, order, family, genus, and species levels

**Figure 9:**
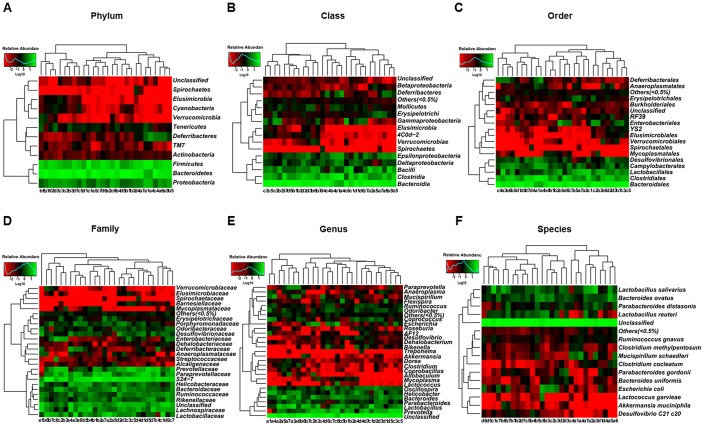
**Heatmaps of gut microbiota composition in pseudo-germ-free mice following transplantation from CD and Non-CD diabetic mice.** (**A**) Heatmap (phylum level). (**B**) Heatmap (class level). (**C**) Heatmap (order level). (**D**) Heatmap (family level). (**E**) Heatmap (genus level). (**F**) Heatmap (species level).

**Figure 10:**
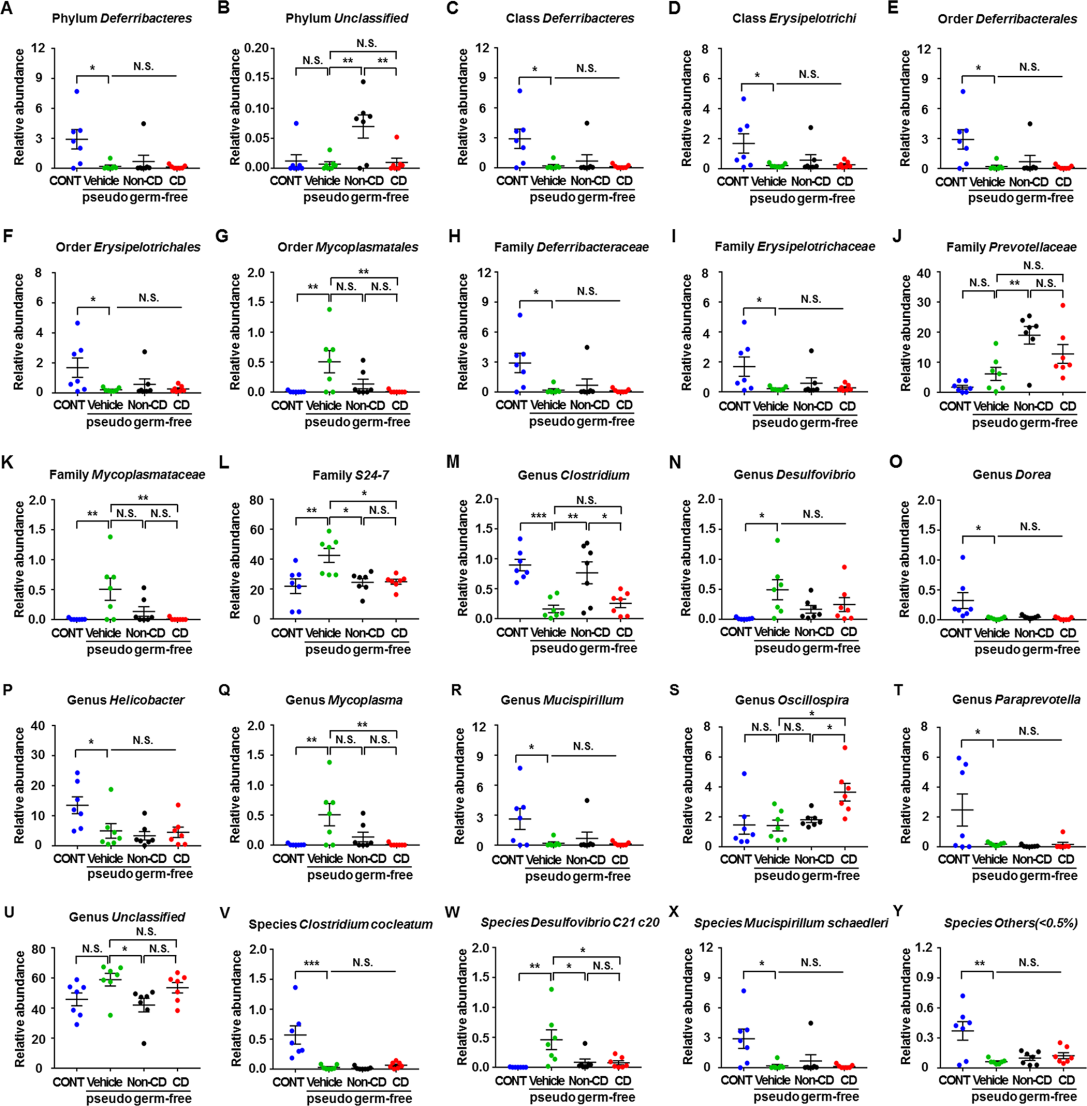
**Differences in relative abundance of various gut microbes among pseudo-germ-free mice following transplantation from CD and Non-CD diabetic mice.** (**A**–**Y**) Relative abundance of (**A**) phylum *Deferribacteres* (one-way ANOVA; F_3,24_ = 5.031, *p* < 0.01), (**B**) phylum *Unclassified* (one-way ANOVA; F_3,24_ = 6.608, *p* < 0.01), (**C**) class *Deferribacteres* (one-way ANOVA; F_3,24_ = 5.031, *p* < 0.01), (**D**) class *Erysipelotrichi* (one-way ANOVA; F_3,24_ = 3.345, *p* < 0.05), (**E**) order *Deferribacterales* (one-way ANOVA; F_3,24_ = 5.031, *p* < 0.01), (**F**) order *Erysipelotrichales* (one-way ANOVA; F_3,24_ = 3.345, *p* < 0.05), (**G**) order *Mycoplasmatales* (one-way ANOVA; F_3,24_ = 5.457, *p* < 0.01), (**H**) family *Deferribacteraceae* (one-way ANOVA; F_3,24_ = 5.031, *p* < 0.01), (**I**) family *Erysipelotrichaceae* (one-way ANOVA; F_3,24_ = 3.345, *p* < 0.05), (**J**) family *Prevotellaceae* (one-way ANOVA; F_3,24_ = 9.825, *p* < 0.001), (**K**) family *Mycoplasmataceae* (one-way ANOVA; F_3,24_ = 5.457, *p* < 0.01), (**L**) family *S24-7* (one-way ANOVA; F_3,24_ = 6.543, *p* < 0.01), (**M**) genus *Clostridium* (one-way ANOVA; F_3,24_ = 10.32, *p* < 0.001), (**N**) genus *Desulfovibrio* (one-way ANOVA; F_3,24_ = 3.552, *p* < 0.05), (**O**) genus *Dorea* (one-way ANOVA; F_3,24_ = 4.82, *p* < 0.01), (**P**) genus *Helicobacter* (one-way ANOVA; F_3,24_ = 4.677, *p* < 0.05), (**Q**) genus *Mycoplasma* (one-way ANOVA; F_3,24_ = 5.457, *p* < 0.01), ® genus *Mucispirillum* (one-way ANOVA; F_3,24_ = 3.575, *p* < 0.05), (**S**) genus *Oscillospira* (one-way ANOVA; F_3,24_ = 5.053, *p* < 0.01), (**T**) genus *Paraprevotella* (one-way ANOVA; F_3,24_ = 4.656, *p* < 0.05), (**U**) genus *Unclassified* (one-way ANOVA; F_3,24_ = 3.379, *p* < 0.05), (**V**) species *Clostridium cocleatum* (one-way ANOVA; F_3,24_ = 12.09, *p* < 0.001), (**W**) species *Desulfovibrio C21 c20* (one-way ANOVA; F_3,24_ = 5.486, *p* < 0.01), (**X**) species *Mucispirillum schaedleri* (one-way ANOVA; F_3,24_ = 3.575, *p* < 0.05), and (**Y**) species *Others (<0.5%)* (one-way ANOVA; F_3,24_ = 7.748, *p* < 0.001). Data are shown as mean ± SEM (n = 7 individual samples/group). *P < 0.05, **P < 0.01 or ***P < 0.001. ANOVA: analysis of variance; CD: cognitive dysfunction; CONT: control; N.S.: not significant; SEM: standard error of the mean.

The heat maps of fecal microbiota composition at the phylum, class, order, family, genus, and species levels revealed substantial differences among control hosts and hosts receiving vehicle, CD mouse fecal bacteria, or Non-CD mouse fecal bacteria (Figure 9A–9F). Overall, 25 bacteria at 6 levels significantly differed among the 4 groups (Figure 10A–10Y). Vehicle-treated pseudo-germ-free mice showed a significant difference in the levels of 21 bacteria (Figure 10A, 10C–10I, 10K–10R, 10T, and 10V–10Y). Although CD and Non-CD mouse fecal microbiota transplants failed to elicit changes in the levels of the phylum *Deferribacteres*, class *Deferribacteres*, class *Erysipelotrichi*, order *Deferribacterales*, order *Erysipelotrichales*, family *Deferribacteraceae*, family *Erysipelotrichaceae*, genus *Desulfovibrio*, genus *Dorea*, genus *Helicobacter*, genus *Mucispirillum*, genus *Paraprevotella*, species *Clostridium cocleatum*, species *Mucispirillum schaedleri*, and species *Others* (<0.5%) (Figure 10A, 10C–10F, 10H, 10I, 10N–10P, 10R, 10T, 10V, 10X, and 10Y), transplantation did alter the relative abundances of 10 other bacteria (Figure 10B, 10G, 10J–10M, 10Q, 10S, 10U, and 10W).

## DISCUSSION

It has been reported that STZ injection at 55 mg/kg for 5 consecutive days can induce a type 1 diabetes-like disorder in rodents [20] associated with irreversible damage to pancreatic islet β-cells [21]. In the present study, we observed that STZ injection significantly increased water and food intake as well as blood glucose levels 2 weeks post-treatment, suggesting successful induction of model type I diabetes. In humans, diabetes increases the risks of Alzheimer’s disease, dementia, and other disorders characterized by CD [22]. A higher risk of developing Alzheimer’s disease in patients with diabetes suggests a shared pathogenesis, although the mechanisms remain unknown [23]. The MWM is a commonly used task for assessing spatial learning and memory in models of neurodegenerative disorders [24]. In the present study, diabetic mice could be stratified into CD and Non-CD groups using the hierarchical cluster analysis of MWM performance indices [17]. Previous studies have reported CD in diabetic mice [20, 25]; however, in the present study, we excluded those without CD to distinctly reveal associations with specific diabetes-related changes (in this case, gut microbiota). To the best of our knowledge, this is the first study adopting hierarchical cluster analysis to study STZ-induced CD. The CD mice demonstrated poor performance in terms of escape latency, the number of platform crossing, and time spent in target quadrant compared with both control mice and other diabetic (Non-CD) mice. These findings suggest that hierarchical cluster analysis is an effective approach to investigate diabetes-induced CD and associations with other diabetes-related pathologies.

The human gut harbors >100 trillion microbes. These gut microbes can affect host behavior by influencing nutrient absorption and subsequently modulating metabolism, by regulating immunity, and by altering enteric nervous system function [26–28]. Collectively, these influences may alter cognitive function. Both preclinical investigations and clinical trials have highlighted the vital role of gut microbiota in the gut–brain axis and suggested possible remote regulation of central nervous system function [29]. Our previous study has demonstrated that CD in SAMP8 mice was strongly associated with gut microbiota composition and relative abundance [16]. Further, we have observed a substantial difference in gut microbiota composition among aged mice with CD after surgery and anesthesia [17]. In the present study, we observed no significant difference in α-diversity (consisting of Shannon and Simpson indices) among the control, CD, and Non-CD groups, suggesting little change in bacterial numbers. However, the separation of groups according to β-diversity (PLS-DA and PCoA) suggested that the microbiota composition was significantly altered by diabetes and by CD.

16S rRNA gene sequencing has become a common approach to study gut microbiota composition and relationships with physiological function [30]. A total of 16 gut bacteria were significantly altered in the fecal samples among the groups. The relative abundances of family *Odoribacteraceae* and genus *Odoribacter* were significantly higher in CD than Non-CD mice, whereas the abundances of six bacteria were significantly lower in the CD group than the Non-CD group. Recently, genus *Odoribacter* was reported to be significantly higher in APP/PS1 mice, a rodent model of Alzheimer’s disease, compared with wild type mice [31]; this result is consistent with our findings that elevated *Odoribacter* is associated with CD. As mentioned above, Alzheimer’s disease is considered a special type of diabetes (type 3 diabetes). Therefore, diabetes-induced CD may share pathogenic mechanisms with AD, including abnormal gut microbiota composition.

Although blood biomarkers and brain imaging techniques have greatly improved the diagnosis of neurodegenerative disorders [32], objective indicators for diagnosing diabetes-induced CD are absent. Therefore, diagnosis depends primarily on subjective cognition function scales [33]. We observed that three gut bacteria were positively or negatively correlated with spatial memory in the MWM. In addition, ROC analysis identified family *Porphyromonadaceae* and genus *Parabacteroides* as sensitive indicators of diabetes-induced CD in mice. These bacteria may thus provide noninvasive biomarkers for the diagnosis of diabetes-induced CD, although additional studies are required for validation.

Pseudo-germ-free mice established using large doses of antibiotics are commonly used for fecal microbiota transplant studies [34]. We have previously reported that SAMP8 mice possess a gut microbiota profile distinct from SAMR1 mice and that fecal microbiota transplant from SAMP8 mice further aggravates MWM performance deficits in pseudo-germ-free mice [16]. However, it is unclear whether these deficits are due to changes in gut microbiota profile or to high-dose antibiotics. Nonetheless, fecal microbiota transplant from Non-CD mice, but not from CD mice, reversed the detrimental effects on cognitive function. In contrast, water and food intake as well as blood glucose levels did not show significant changes. These results suggest that diabetes-induced CD may be unrelated to other typical pathogenic processes of diabetes but could be associated with those of Alzheimer’s disease.

16S rRNA gene sequencing revealed that 25 bacteria were altered at 6 levels following fecal microbiota transplant and that these changes were associated with alterations in cognitive function. In addition, the levels of 21 bacteria were significantly altered in vehicle-treated mice, suggesting the effects of antibiotic treatment [35]. *Clostridium*, a relatively novel genus of anaerobic bacteria, has been associated with CD [36]. In the present study, CD mouse fecal microbiota transplant significantly decreased the levels of *Clostridium*, whereas Non-CD mouse fecal microbiota transplant increased *Clostridium* levels. These findings support the notion that regulating gut microbiota composition can improve diabetes-induced CD.

In conclusion, these findings strongly suggest that abnormal gut microbiota composition contributes to diabetes-induced CD. Considering the possible pathogenic link between diabetes and Alzheimer’s disease, the regulation of gut microbiota may be an effective therapeutic target for age-related cognitive disorders.

## MATERIALS AND METHODS

### Animals

Overall, 80 C57BL/6J mice (age, 8 weeks; 20–25 g) were purchased from Beijing Vital River Laboratory Animal Technology (Beijing, China). Animals were housed under controlled temperature (22°C ± 2°C), controlled relative humidity (60% ± 5%), and a 12-h/12-h light/dark cycle with *ad libitum* access to food and water. Animals were allowed to acclimate for a week before experiments. All experimental protocols and animal handling procedures were conducted in strict accordance with the recommendations in the Guide for the Care and Use of Laboratory Animals published by the National Institutes of Health (NIH Publications No. 80-23, revised in 1996). This study was approved by the Experimental Animal Committee of Tongji Hospital, Tongji Medical College, Huazhong University of Science and Technology (Wuhan, China).

### Animal models of type 1 diabetes mellitus

As shown in Figure 1A, animals were randomly divided into two groups after acclimation of 7 days: control (n = 8) and experimental (n = 32). Animals were fasted for 12 h prior to treatment [37]. A freshly prepared solution of 10 mg/mL STZ (Absin Bioscience Inc., Shanghai, China) in 0.1 M sodium citrate buffer (pH 4.5) was used to establish type 1 diabetes models. As previously described [20], mice were intraperitoneally injected with STZ at 55 mg/kg for 5 consecutive days, whereas mice in the control group were injected with the same dose of sodium citrate buffer. Body weight and water and food intake were recorded once a week, and fasting blood glucose levels was assessed every 2 weeks from a tail vein blood sample using a OneTouch® Ultra blood glucose meter. Mice with blood glucose levels >11.1 mmol/L were selected as the diabetes model group for subsequent experiments [38]. In the present study, 26 diabetes model mice were obtained (26 of 32, 81.25%) and used for subsequent experiments. At 8 weeks later, mice were assessed using the MWM for the evaluation of cognitive function.

### Morris water maze analysis

At 8 weeks after STZ injection, spatial learning and memory were assessed using the MWM [39]. The MWM task was performed in a circular pool (diameter 120 cm, height 50 cm) filled with water (23 ± 1 °C) made opaque by the addition of nontoxic titanium white-colored dye. The pool was located in a room with low-level indirect lighting. A white platform (diameter 10 cm) was submerged 0.5–1 cm below the water surface in the target quadrant. Mice were trained to locate the hidden platform by performing four trials per day for 5 consecutive days. After locating the platform, the mouse was allowed to stay on it for 15 s before being removed. If a mouse did not find the platform within 60 s, it was gently guided to the platform and allowed to stay for 15 s. For all training trials, time and distance to reach the platform (escape latency and path length) were recorded. A probe test was conducted immediately after the 5-day period to evaluate spatial memory. During the probe test, the platform was removed from the pool and mice were allowed to swim freely for 60 s in any quadrant. The number of platform crossings and time spent in each quadrant were recorded.

### Pseudo-germ-free mice modeling

The pseudo-germ-free mouse model was established based on a previous study with slight modification [40]. Briefly, broad-spectrum antibiotics (ampicillin 1 g/L, neomycin sulfate 1 g/L, metronidazole 1 g/L, Sigma-Aldrich Co. Ltd, USA) were dissolved in drinking water and administered *ad libitum* to C57BL/6 mice for 14 consecutive days. The drinking solution was renewed every 2 days.

### Fecal microbiota transplantation

Diabetes model mice were individually placed in a clean cage containing sterilized filter paper. Fecal samples were collected immediately after defecation in a sterilized centrifuge tube. The filter paper was replaced for each mouse. Feces were stored in a −80°C freezer until analysis and transplantation [41]. Fecal microbiota was prepared by diluting 1 g of fecal sample obtained from CD or Non-CD mice in 10 mL of sterile saline. The fecal material was suspended and 0.2 mL of the suspension was administered by gavage to each recipient pseudo-germ-free mouse for 14 consecutive days [40].

### 16S rRNA gene sequencing of fecal samples

Fecal samples were collected after all behavioral tests (Figure 1A and Figure 4A), placed in 1.5 ml tubes, snap frozen on dry ice, and stored at −80° C prior to 16S rRNA gene sequencing at Beijing Genomics Institute (Shenzhen, China). DNA extraction was performed using TIANamp stool DNA kits (Tiangen Biotechnology Company, Beijing, China). Thereafter, genomic DNA was amplified in 50 μL triplicate reactions with the following primers specific to the V3−V4 region of the bacterial 16S rRNA gene: 338F (5′-ACTCCTACGGGAGGCAGC-3′) and 806R (5′-GG ACTACHVGGGTWTCTAAT-3′). The reverse primer contained a sample barcode and both primers were connected with an Illumina sequencing adapter. Polymerase chain reaction (PCR) products were purified, and the concentrations were adjusted for sequencing on an Illumina Miseq PE300 system. The original sequencing reads from the samples were sorted based on the unique barcodes, and the barcodes, linkers, and PCR primer sequences were then removed. The resultant sequences were screened for quality and ≥70 base pairs were selected for bioinformatics analysis. All sequences were classified using the National Center for Biotechnology Information BLAST and SILVA databases. Distance calculation, operational taxonomic unit clustering, rarefaction analysis, and estimator calculation (α-diversity and β-diversity) were performed using the MOTHUR program [42].

### Receiver operating characteristic curve analyses

ROC curves illustrate the diagnostic ability of a binary classifier system with the true positive rate (sensitivity) as the ordinate and the false positive rate (1-specificity) as the abscissa. The ROC curves were used to distinguish mice with diabetes-induced CD from all other mice. The value of the area under the curve (AUC) represents the accuracy of the diagnosis.

### Statistical analysis

Data are presented as the mean ± standard error of the mean (SEM). All statistical analyses were performed using GraphPad Prism 7 (GraphPad Software, San Diego, CA, USA). For the hierarchical cluster analysis of MWM performance indices to define CD and Non-CD groups, the data were first standardized as z scores. Hierarchical cluster analysis was performed using Ward’s method. Correlation analysis was conducted using Pearson’s product-moment coefficient. The diagnostic cutoff value, sensitivity, specificity, and accuracy of each bacterium were determined using ROC curve analysis. Group means were compared by one-way or two-way analysis of variance (ANOVA), followed by post hoc Tukey’s tests for pair-wise comparisons. A P < 0.05 (two-tailed) was considered significant for all tests.
